# Cryopreservation of Human Midbrain Dopaminergic Neural Progenitor Cells Poised for Neuronal Differentiation

**DOI:** 10.3389/fcell.2020.578907

**Published:** 2020-11-05

**Authors:** Nicola J. Drummond, Karamjit Singh Dolt, Maurice A. Canham, Peter Kilbride, G. John Morris, Tilo Kunath

**Affiliations:** ^1^MRC Centre for Regenerative Medicine, Institute for Stem Cell Research, School of Biological Sciences, The University of Edinburgh, Edinburgh, United Kingdom; ^2^Cytiva, Cambridge, United Kingdom; ^3^UK Centre for Mammalian Synthetic Biology, The University of Edinburgh, Edinburgh, United Kingdom

**Keywords:** human embryonic stem cells, dopaminergic differentiation, midbrain dopaminergic neurons, cryopreservation, Parkinson’s disease

## Abstract

Human pluripotent stem cells can be differentiated into midbrain dopaminergic (mDA) neurons by directing cells through a floor plate progenitor stage. The developmental identity of mDA neurons produced using floor plate protocols is similar to *substantia nigra* neurons, and this has improved the ability to model Parkinson’s disease (PD) in a dish. Combined with the unlimited growth potential of pluripotent stem cells, mDA neural progenitor cell production can provide a scalable source of human dopaminergic (DA) neurons for diverse applications. However, due to the complexity and length of the protocols and inherent differences between cell lines, considerable variability of the final population of neurons is often observed. One solution to this problem is to cryopreserve committed mDA neural progenitor cells in a ready-to-use format. Creating a bank of cryopreserved mDA neural progenitor cells poised for neuronal differentiation could significantly improve reproducibility and facilitate collaborations. Here we have compared six (6) different commercial cryopreservation media and different freezing conditions for mDA neural progenitor cells differentiated from human embryonic stem cell (hESC) lines. Significant differences in cell recovery were observed at 24 h post-thawing, but no differences were observed immediately upon thawing. The presence of ROCK inhibitors improved cell recovery at 24 h for all cryopreservation media tested. A faster cooling rate of 1–2°C/min was significantly better than 0.5°C/min for all conditions tested, while rapid thawing at 37°C was not always superior to slow thawing at 4°C. Importantly, cryopreservation of mDA neural progenitor cells did not alter their potential to resume differentiation into mDA neurons. Banks of cryopreserved committed mDA neural progenitor cells provide a method to generate human DA neurons with reduced batch-to-batch variability, and establish a mechanism to share lineage-primed cells for collaborative research.

## Introduction

Parkinson’s disease (PD) is a common neurodegenerative condition characterized by progressive and selective neuronal cell loss. Although the subtypes of neurons that become dysfunctional and die in PD are diverse, the dopaminergic (DA) neurons of the *substantia nigra* are particularly affected in this condition. The embryological origin of nigral DA neurons is a population of radial glial-like cells in the floor plate of the mesencephalon (Ono et al., 2007; Bonilla et al., 2008). Significant progress has been made in the last 10 years to produce floor plate cells and authentic midbrain DA (mDA) neurons from human embryonic stem cells (hESCs) and induced pluripotent stem cells (iPSCs) (Fasano et al., 2010; Kriks et al., 2011; Kirkeby et al., 2012; Xi et al., 2012). Single-cell RNAseq of hESC/iPSC-derived mDA neurons generated by the floor plate protocol showed significant overlap with multiple human fetal mDA cell types (La Manno et al., 2016). The functionality of mDA neurons differentiated from hESCs and iPSCs has been extensively investigated in *in vivo* pre-clinical animal models of PD (Kriks et al., 2011; Kirkeby et al., 2012; Kikuchi et al., 2017). hESC-derived mDA neural progenitor cells, upon transplantation, could rescue the DA deficit in the rat 6-hydroxydopamine (6-OHDA) lesion model of PD and were demonstrated to be functionally equivalent to human fetal ventral midbrain tissue (Grealish et al., 2014). Furthermore, human iPSC-derived mDA neural progenitor cells, FACS-sorted for the floor plate marker CORIN, could rescue a macaque model of PD established by 1-methyl-4-phenyl-1,2,3,6-tetrahydropyridine (MPTP) lesion (Kikuchi et al., 2017). The improved mDA differentiation protocols have also enhanced the ability to model aspects of PD in a dish, including recapitulating neuronal synucleinopathy (Chen et al., 2019), and investigation of DA electrophysiology (Chen et al., 2020).

Differentiation of hESCs/iPSCs into mDA neurons is a complex and multi-stage process, and it is known that different iPSC lines from the same patient can have significantly different propensities to produce mDA neurons (Devine et al., 2011). Furthermore, the positional identity of floor plate cells produced from hESCs/iPSCs is highly sensitive to small changes in WNT signaling (Kirkeby et al., 2012). A cryopreserved mDA neural progenitor cell bank could provide a quality-controlled population of cells from which mDA neuronal differentiation and maturation can be conducted. This will reduce variability across experiments, and facilitate collaborations across multiple laboratories.

Cryopreservation of primary rat fetal mesencephalic tissue resulted in a greater than 50% loss of viability compared to non-frozen cells, but the surviving neurons, when grafted into the rat 6-OHDA lesion model, were able to ameliorate the amphetamine-induced rotation phenotype (Sauer et al., 1992). However, attempts to cryopreserve human fetal mesencephalic tissue prior to functional assessment in the 6-OHDA lesion model were less successful with more than 90% loss of viable mDA cells compared to non-frozen controls, and no significant rescue of amphetamine-induced rotations (Frodl et al., 1994). More recently, successful cryopreservation of hESC/iPSC-derived mDA cells using a floor plate protocol has been reported (Niclis et al., 2017; Leitner et al., 2019). Furthermore, commercial cryopreserved human iPSC-derived mDA cells (iCell DopaNeurons) have been directly transplanted into rat and non-human primate lesion models of PD (Wakeman et al., 2017). Thawed mDA neural progenitor cells could rescue amphetamine-induced rotations after transplantation into the rat 6-OHDA lesion model, and survival and maturation into mDA neurons was observed in the MPTP-lesion monkey model (Wakeman et al., 2017). However, optimization of the cryopreservation process has not been reported nor has there been a systematic investigation to evaluate cryopreservation conditions for mDA neural progenitor cells. Here we investigate different cryopreservation conditions for human mDA neural progenitor cells, present the first report comparing multiple commercial cryopreservation media, and propose guidelines for best practices to optimize cryopreservation of human ESC/iPSC-derived cell products.

## Materials and Methods

### Human Embryonic Stem Cell Culture

Approval for the use of MasterShef7 (MShef7) and RC17 hESCs was granted by the MRC Steering Committee for the UK Stem Cell Bank and for the Use of Stem Cell Lines (ref. SCSC13-18 for MShef7 and ref. SCSC13-19 for RC17). RC17 hESC line, kindly provided by Roslin Cells Limited, was derived under Good Manufacturing Production (GMP) conditions (De Sousa et al., 2016). MShef7 hESC line, kindly provided by Prof Harry Moore, was derived in GMP conditions at the University of Sheffield. RC17 and MShef7 lines have normal female and male karyotypes, respectively, and both carry a single naturally-occurring large copy number variation (>100 kb) that was most likely present in the donated blastocyst (Canham et al., 2015). hESCs were maintained in StemMACS^TM^ iPS-Brew XF (iPS-B, Miltenyi Biotec) on Laminin-521-coated plates (L521, 5 μg/ml, Biolamina). Once hESCs reached 70–90% confluency, they were passaged as clumps using EDTA (0.5 mM, Thermo Fisher Scientific).

### Midbrain Dopaminergic Differentiation

Self-renewing hESCs were lifted with EDTA (0.5 mM), counted, and plated for differentiation at 40,000 cells/cm^2^ on Laminin-111-coated plates (L111, 5 μg/ml, Biolamina) in 50% Neurobasal^TM^ medium (Thermo Fisher Scientific), 50% DMEM/F12 (Thermo Fisher Scientific), B27 (1:50, Thermo Fisher Scientific), N2 (1:100, Thermo Fisher Scientific), L-glutamine (2 mM, Thermo Fisher Scientific), Sonic hedgehog (SHH-C24II, 600 ng/ml, R&D), CHIR99021 (0.9 or 1 μM, Miltenyi Biotec), SB431542 (10 μM, Tocris), LDN193189 (100 nM, Stemgent), and Y27632 (10 μM, Tocris). The culture medium was exchanged on day 2 with the above medium without Y27632. On day 4 and day 7, cells were fed with medium consisting of 50% Neurobasal, 50% DMEM/F12, B27 (1:100), N2 (1:200), L-glutamine (2 mM), SHH-C24II (600 ng/ml), CHIR99021 (0.9 or 1 μM), SB431542 (10 μM), and LDN193189 (100 nM). On day 9, cells were fed with the above medium supplemented with FGF8b (100 ng/ml, R&D) and heparin (1 μg/ml, Sigma). On day 11, cells were lifted using Accutase (Sigma) and re-plated at 800,000 cells/cm^2^ on L111-coated plates in Neurobasal medium supplemented with B27 (1:50), L-glutamine (2 mM), BDNF (20 ng/ml, Peprotech), GDNF (10 ng/ml, Peprotech), ascorbic acid (0.2 mM, Sigma), FGF8b (100 ng/ml), heparin (1 μg/ml), and Y27632 (10 μM). Cells were fed on day 14 with day 11 medium without Y27632. On day 16, cells were lifted with Accutase and either (i) cryopreserved or (ii) re-plated at 800,000 cells/cm^2^ on L111-coated plates in Neurobasal medium with B27 (1:50), L-glutamine (2 mM), BDNF (20 ng/ml), GDNF (10 ng/ml), ascorbic acid (0.2 mM), dibutyryl cyclic AMP (db-cAMP, 0.5 mM, Sigma), DAPT (1 μM, Tocris), and Y27632 (10 μM). From day 18 onward, medium was exchanged every 2–3 days. A detailed version of this protocol is available at: dx.doi.org/10.17504/protocols.io.bddpi25n.

### Cryopreservation

At day 16 of differentiation, cells were lifted using Accutase and counted using TC20^TM^ Automated Cell counter (BioRad). Cells were centrifuged (300 *g*, 3 min) and resuspended in the appropriate freezing medium (Table 1) at 1.02 × 10^7^ cells/ml and 100 μl of cell suspension was added to each 0.5-ml cryovial (FluidX). Cells were frozen in a VIA Freeze^TM^ Duo controlled-rate freezer (Cytiva, Cambridge, United Kingdom) using the following protocol: 4°C hold for 10 min and then temperature was reduced at 0.5, 1, or 2°C/min until −80°C was reached. Cryovials were transported on dry ice and transferred to vapor phase liquid nitrogen (∼−170°C) for long term storage.

**TABLE 1:** List of media used for cryopreservation.

Cryopreservation medium	Abbreviation	DMSO content (v%)	Supplier
STEM-CELLBANKER^®^	SCB	10% DMSO	Amsbio
Synth-a-Freeze^TM^ Medium	SYF	10% DMSO	Thermo Fisher
PSC Cryopreservation Medium	PSC	10% DMSO	Thermo Fisher
CryoStor^®^ CS10 Freeze Medium	CS10	10% DMSO	BioLife Solutions
CryoStor^®^ CS5 Freeze Medium	CS5	5% DMSO	BioLife Solutions
Cellvation Cryopreservation Medium	CV	DMSO-free	MP Biomedicals
**Hibernation medium**			
HypoThermosol^®^	Hypo	DMSO-free	BioLife Solutions

### Thawing and Cell Counting

Cells were thawed by exposing the vial to 37°C water for 1–2 min (rapid thawing) or by placing the vial in air at 4°C for approximately 10 min (slow thawing). The cell suspension was removed from the cryovial and placed onto 1 ml Neurobasal^TM^ medium with B27 (1:50) and L-glutamine (2 mM) and centrifuged (300 *g*, 3 min). The supernatant was removed and the cell pellet was resuspended in 300 μl of day 16 differentiation medium supplemented with either Y27632 (10 μM) or RevitaCell^TM^ (1X, Thermo Fisher Scientific). An aliquot of cells was taken for cell counting (0 h cell count) prior to plating in L111-coated 48-well plates (Corning); 24 h later, the conditioned medium was collected and combined with a DPBS wash of the cells to collect all the floating cells. This was centrifuged (300 *g*, 3 min) and cells were resuspended in a small volume (20–120 μl) of Neurobasal medium and counted in the presence of Trypan Blue using the TC20^TM^ Automated Cell counter (Bio-Rad) (24 h floating cell count). The adherent cells were lifted with Accutase and similarly counted (24 h attached cell count).

### Immunofluorescence Staining and Image Quantification

Cells were fixed with 4% formaldehyde for 20 min and washed three times in PBS. Blocking buffer (0.1% Triton X-100, 2% goat serum or donkey serum in PBS) was added to fixed cells for 30 min. Primary antibodies TH (1:1000, rabbit, Millipore), β-III tubulin (1:1000, mouse IgG2a, R&D), LMX1A (1:2000, rabbit, Millipore), FOXA2 (1:100, goat, Santa Cruz), EN1 (1:50, rabbit, GeneTex), and CORIN (1:1000, rat, R&D) were incubated with fixed cells overnight at 4°C and then washed three times in PBS with 0.1% Triton X-100. Secondary antibodies (Thermo Fisher Scientific) in blocking buffer were incubated with cells for 2 h in the dark at room temperature. They were washed three times in PBS with Triton X-100 and incubated with DAPI (10 μg/ml, Thermo Fisher Scientific) before imaging on an Olympus IX51 inverted microscope. Quantification of TH and β-III tubulin immunostaining was performed with Fiji software (Schindelin et al., 2012). Briefly, RGB images were converted to 8-bit grayscale, and then manually thresholded during conversion to binary images. Efficiency of DA neuronal differentiation was estimated by calculating the ratio of total TH to total β-III tubulin immunostaining for each image.

### FACS

Human embryonic stem cells and cells at day 16 of differentiation were lifted with Accutase, centrifuged (2150 *g*, 1.5 min), and resuspended in FACS Buffer (PBS + 2% FBS) with CORIN antibody (1:200, rat, R&D), and incubated for 15 min on ice. A rat IgG isotype antibody was used for the control FACS. Cells were then centrifuged and washed in FACS Buffer and incubated on ice (15 min) with secondary antibody donkey anti-rat IgG Alexa Fluor-488 (Thermo Fisher Scientific). Cells were centrifuged and washed in FACS Buffer and flow cytometry data were collected using the FACS Calibur (BD Biosciences) and post-acquisition analysis was performed using FlowJo software.

### RT-qPCR

RNA extraction was performed using the MasterPure^TM^ Complete DNA and RNA Purification Kit (Epicenter, MC85200), according to manufacturer’s instructions. RNA concentration was quantified using a NanoDrop spectrophotometer. Total RNA (1 μg in 10 μl) was used for cDNA synthesis. The samples were incubated with 1 μl dNTP mix (10 mM, Thermo Fisher Scientific) and 1 μl Random Primer Mix (60 μM, NEB) at 65°C for 5 min and then chilled on ice. After a brief centrifugation, 4 μl 5x First-strand buffer (Thermo Fisher Scientific), 2 μl DTT (0.1 M, Thermo Fisher Scientific), and 1 μl RNaseOUT (40 units/μl, Thermo Fisher Scientific) were added. The contents were incubated at 37°C for 2 min before 1 μl M-MLV reverse transcriptase (200 units/μl, Thermo Fisher Scientific) was added. This was incubated at room temperature for 10 min and then 37°C for 1 h. The reaction was inactivated at 90°C for 10 min. qPCR was performed using the Roche LightCycler^®^ 480 System with the Universal Probe Library (UPL) (Roche). The Roche UPL Assay design center was used to design forward (F) and reverse (R) intron-spanning primers with a specific UPL probe for each gene (*TBP* F-gaacatcatggatcagaacaaca R-atagggattccgggagtcat, Probe 87; *LMX1A* F-tggaggagaacttccaaagc R-cagacagacttggggctcac, Probe 3; *FOXA2* F-gggtgattgctggtcgttt R-atactggaagccgagtgcat, Probe 5; *EN1* F-gcacacgttattcggatcg R-gcttgtcctccttctcgttc, Probe 88; *NURR1* F-atttcctcgaaaacgcctgt R-catactgcgcctgaacacaa, Probe 41; *PITX3* F-tgtcagacgctggcactc R-ccgaggccttttctgagtc, Probe 24; *TH* F-gattccccgtgtggagtaca R-aagcaaaggcctccaggt, Probe 12). Reactions (10 μl) containing primers, UPL Probe, LightCycler^®^ 480 Probes Master mix (Roche), and PCR water were performed in 384-well plates as described in the manufacturer’s instructions. The results were normalized to transcript levels of TATA-binding protein (*TBP*).

## Results

**FIGURE 1:**
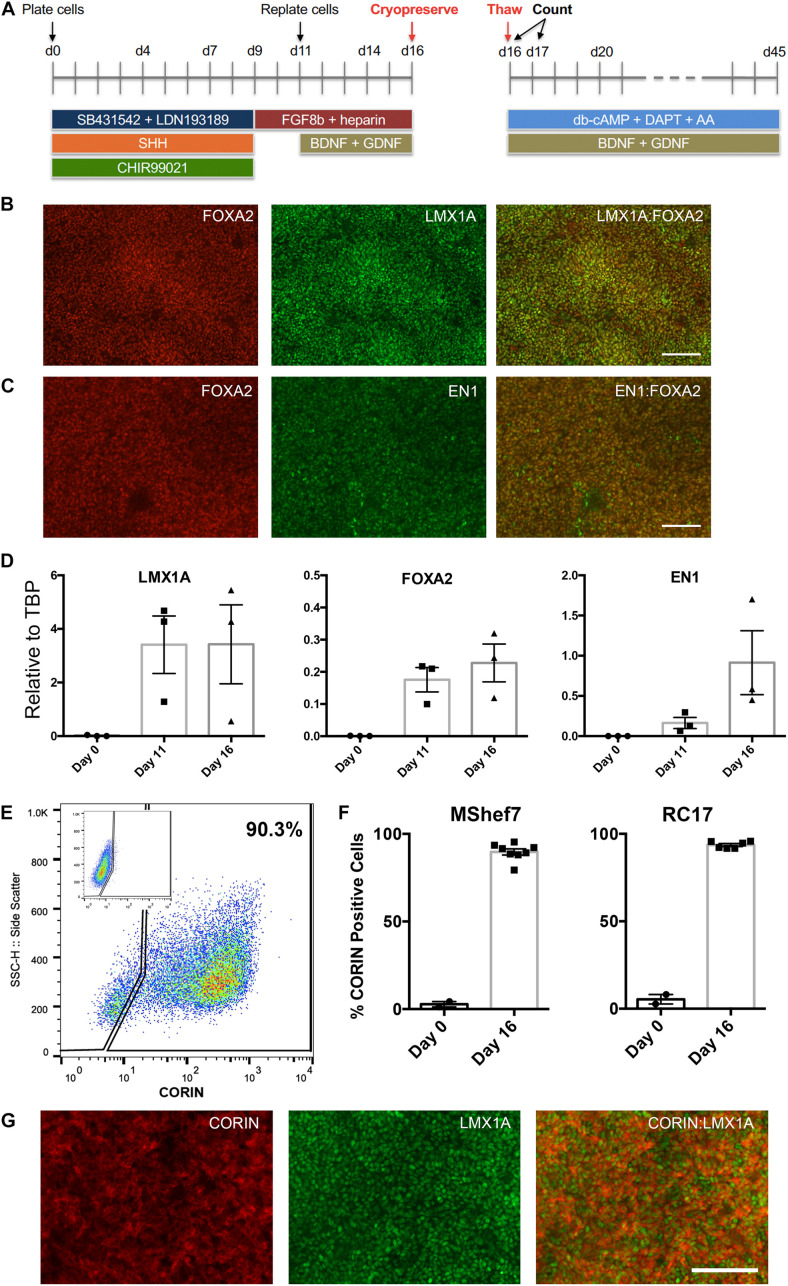
Differentiation of human embryonic stem cells (hESCs) into midbrain dopaminergic (mDA) precursors for cryopreservation. **(A)** Schematic of the mDA differentiation protocol. hESCs are plated as clumps on day 0 in the presence of dual SMAD inhibitors, SB431542 + LDN193189, Sonic hedgehog (SHH), and the GSK3β inhibitor, CHIR99021. At day 9, the medium is switched to contain only FGF8b and the co-factor heparin, and cells are lifted and re-plated at day 11 when the neurotrophic factors BDNF and GDNF are added. At day 16, the cells are lifted and counted for cryopreservation. Live/dead cell counts were performed immediately upon thawing and at 24 h after thawing (day 17). mDA neuronal maturation was conducted in the presence of BDNF, GDNF, db-cAMP, ascorbic acid (AA), and the Notch inhibitor DAPT, up to day 45. Co-immunostaining for midbrain floor plate markers, FOXA2 with LMX1A **(B)**, and FOXA2 with EN1 **(C)**, at day 16 revealed near homogenous expression at the point of cryopreservation. Scale bar, 120 μm. **(D)** RT-qPCR analysis of *LMX1A*, *FOXA2*, and *EN1* expression relative to TBP at Day 0, 11, and 16 of differentiation for three independent experiments (*n* = 3). **(E)** FACS analysis for CORIN expression at day 16 showed the majority of cells expressed this ventral floor plate marker. The control FACS with rat isotype control antibody is shown in the inset. **(F)** Percentage CORIN-positive cells as determined by FACS for undifferentiated day 0 MShef7 hESCs (*n* = 2) and RC17 hESCs (*n* = 2), and day 16 MShef7-derived (*n* = 8) and RC17-derived (*n* = 6) mDA neural progenitor cells. **(G)** Co-immunostaining of day 16 MShef7-derived mDA neural progenitor cells for CORIN and LMX1A. Scale bar, 120 μm.

Two clinical-grade hESC lines, MasterShef7 (MShef7) and RC17, were chosen for this study since they efficiently differentiated into mDA neural progenitor cells and into mDA neurons using a modified 2D floor plate protocol (Kriks et al., 2011; Kirkeby et al., 2017). Briefly, hESCs were plated onto a matrix of Laminin-111 in the presence of dual-Smad inhibition (SB431542 + LDN193189), 0.9–1 μM GSK3β inhibitor (CHIR99021), and 600 ng/ml Sonic hedgehog (SHH-C24II). FGF8b and heparin were added at day 9 of differentiation, and brain derived neurotrophic factor (BDNF) and glial cell derived neurotrophic factor (GDNF) were added at day 11 (Figure 1A). The differentiating mDA neural progenitor cells can be conveniently cryopreserved at day 11 or day 16 of differentiation, since these are time-points when the cells are lifted and re-plated in the protocol. However, for all experiments in this study, cells were frozen at day 16 since cells at this stage of maturity can be transplanted into pre-clinical rat models of Parkinson’s (Kirkeby et al., 2012), and no further re-plating steps are required to produce mature mDA neurons. In order to examine the *in vitro* differentiation potential of the cryopreserved cells, the mDA neural progenitor cells are thawed onto Laminin-111 in the presence of neurotrophic and neuronal maturation factors to resume differentiation into mDA neurons up to day 45 (Figure 1A). At the point of freezing (day 16), the vast majority of cells expressed the mDA markers, LMX1A, FOXA2, and Engrailed1 (EN1) as determined by immunocytochemistry (Figures 1B,C), and transcripts for these three transcription factors increased significantly during the differentiation process (Figure 1D). Flow cytometry for CORIN, a ventral floor plate marker, demonstrated that MShef7 and RC17 hESCs routinely produced ∼90% CORIN-positive cells by day 16 of differentiation (Figures 1E,F). Furthermore, immunocytochemistry at day 16 revealed that LMX1A-positive cells exhibited cytoplasmic or membrane immunostaining for CORIN (Figure 1G).

**FIGURE 2:**
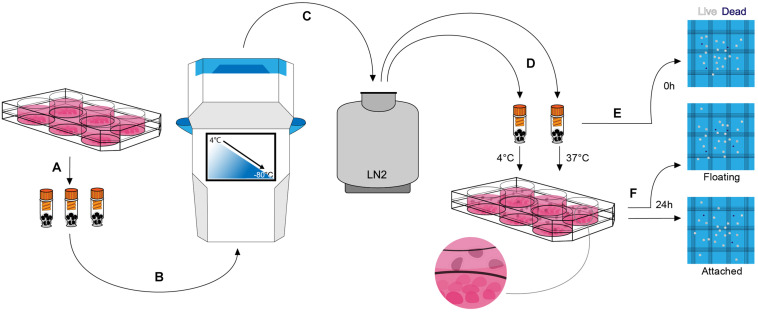
Cryopreservation experimental procedure. **(A)** At day 16 of mDA differentiation, cells were lifted, counted, and resuspended in a clinical-grade cryopreservation medium prior to transferring to FluidX cryovials. **(B)** Cryovials were placed in the VIA Freeze controlled-rate freezer and cells were cryopreserved at a constant rate of cooling between 0.5 and 2°C/min to a final temperature of –80°C. **(C)** Cryovials were transferred to the vapor phase of liquid nitrogen for long term storage. **(D)** Cryopreserved cells were thawed at 4 or 37°C. **(E)** A live/dead cell count was performed immediately after thawing (0 h count) prior to plating the cells. **(F)** 24 h after plating cells (day 17 of differentiation) the non-adherent floating cells and the adherent attached cells were subjected to live/dead cell counts (“24 h—Floating” count and “24 h—Attached” count, respectively).

Human embryonic stem cell-derived mDA neural progenitor cells were cryopreserved at a density of ∼1 × 10^7^ cells/ml in a total volume of 100 μl in six (6) different clinical-grade cryopreservation media that are commercially available (Table 1). This relatively small volume was used to facilitate uniform thawing and the high cell density was published to improve cell viability when compared to cells frozen at ∼1 × 10^6^ cells/ml (Terry et al., 2010). The media investigated included (i) STEM-CELLBANKER^®^ (SCB), (ii) Synth-a-Freeze^TM^ Medium (SYF), (iii) PSC Cryopreservation Medium (PSC), (iv) CryoStor^®^ CS10 Freeze Medium, (v) CryoStor^®^ CS5 Freeze Medium, and (vi) Cellvation Cryopreservation Medium (CV). CS5 contained 5% DMSO (v/v), while CV is a DMSO-free cryopreservation medium. The other four cryopreservation media contained 10% DMSO (v/v). HypoThermosol^®^, a 4°C DMSO-free hibernation medium, was used as a negative control for these experiments. Cooling rates were varied from 0.5 to 2°C/min using a VIA Freeze^TM^ Duo controlled-rate freezer starting from 4°C and stopping at −80°C before transferring vials to vapor phase liquid nitrogen storage (Figure 2). The vapor phase of liquid nitrogen (below −170°C) was chosen since this is below the glass transition temperature of cells in cryoprotectant (∼−120°C), and biological material is known to be highly stable at this temperature with no changes in cell viability for over 1 year (Silani et al., 1988; Massie et al., 2013; Meneghel et al., 2019). The recovery of mDA neural progenitor cells was performed by either thawing vials slowly in air at 4°C or rapidly in a water-bath set to 37°C. Cell viability was assessed by Trypan blue exclusion immediately upon thawing prior to plating in mDA neuronal differentiation conditions, and again at 24 h post-thawing. The latter cell viability test was performed since cryopreservation-induced apoptosis is reported to occur in some cell types 12–24 h after thawing (Baust et al., 2001). In order to gain a more detailed account of the cell population, both floating cells and attached cells were counted at 24 h post-thawing (Figure 2). However, even this will not capture the fate of all cells, since some cryopreserved cells will be mechanically destroyed during the freezing and thawing process due to irreversible intracellular ice crystal formation (Murray and Gibson, 2020). These destroyed cells will be missing from the live/dead and attached/floating cell counts. For this reason, we calculate both cell viability and cell recovery, the latter defined as a percentage of cells initially frozen.

**FIGURE 3:**
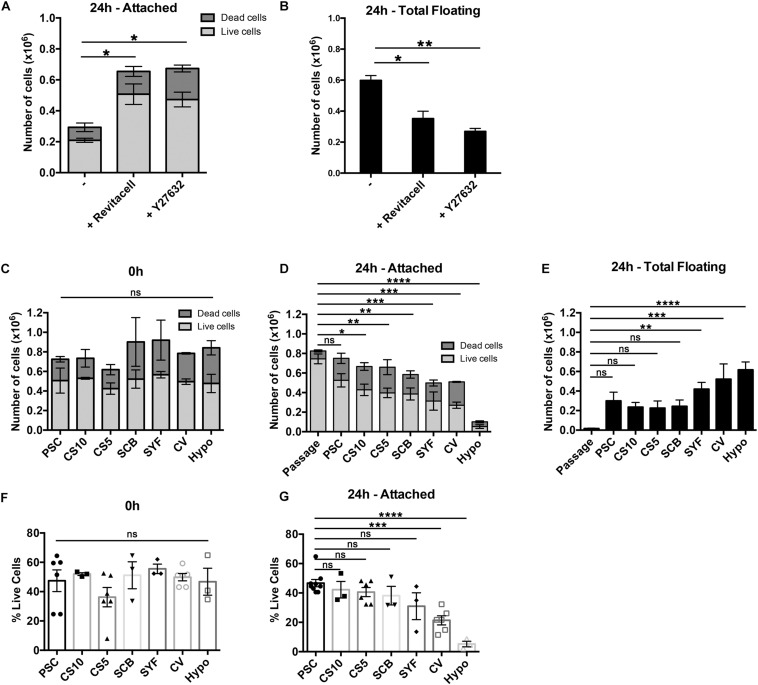
Assessment of cell viability after cryopreservation in the presence or absence of ROCK inhibitors, and using seven different clinical-grade media. **(A,B)** Cell viability of mDA cells after cryopreservation in the absence of ROCK inhibitors or in the presence of RevitaCell^TM^ or Y27632. **(A)** Live/dead *attached* cell numbers at 24 h after thawing, and **(B)** total number of *floating* cells at 24 h (*n* = 2 experimental replicates). **(C)** Comparison of cell viability after cryopreservation in PSC Cryopreservation Medium (PSC), CryoStor^®^ CS10 Freeze Media (CS10), CryoStor^®^ CS5 Freeze Media (CS5), STEM-CELLBANKER^®^ (SCB), Synth-a-Freeze Medium (SYF), Cellvation Cryopreservation Medium (CV), and Hypothermosol^®^ (Hypo) immediately after thawing. At 24 h post-thawing live/dead cell counts of the attached cells **(D)** and total floating cells **(E)** were determined (*n* = 3 experimental replicates), and compared to freshly passaged cells (*n* = 6 experimental replicates). Live cells as a percentage of initial cell number frozen immediately upon thawing **(F)**, and attached cells at 24 h post-thawing **(G)** (*n* = 3 experimental replicates). ns, not significant, *p < 0.05, **p < 0.01, ***p < 0.001, ****p < 0.0001 one-way ANOVA with Tukey’s multiple comparisons test.

Rho-associated kinase (ROCK) inhibitors improved viability of single hESCs upon passaging and after cryopreservation (Watanabe et al., 2007), and they significantly improved the recovery of cryopreserved hESC-derived cardiomyocytes (Kim et al., 2011). Therefore, we first compared Y27632 (10 μM), a cell-permeable ROCK inhibitor, with the commercial formulation, RevitaCell^TM^, which contains a ROCK inhibitor and antioxidants. At 24 h post-thawing both RevitaCell^TM^ and Y27632 significantly increased the number of attached live mDA neural progenitor cells (Figure 3A), and significantly reduced the number of floating cells (Figure 3B). There were no significant differences between RevitaCell^TM^ and Y27632 by these measures. Since RevitaCell^TM^ is manufactured at clinical-grade for medical devices (21 CFR Part 820 and ISO 13485), it was used for all subsequent experiments.

We conducted a side-by-side comparison of six commercially-available, clinical-grade cryopreservation media, and a DMSO-free hibernation medium, HypoThermasol^®^ (Hypo) (Table 1). Cell viability immediately upon thawing was not significantly different for all cryopreservation media, nor was it significantly different to the hibernation medium (Figure 3C). However, at 24 h post-thawing, there were significant differences in cell viability for the different cryopreservation media tested. Cells frozen in the hibernation medium, Hypo, gave the lowest level of cell viability, while PSC Cryopreservation Medium was the only one that was not significantly different from freshly passaged cells (Figure 3D). Cellvation, a DMSO-free cryopreservation medium, was not significantly worse than the DMSO-containing cryopreservation media, while Hypo was significantly worse than PSC, CS10, CS5, and SCB cryopreservation media (Supplementary Table S2). The number of floating cells at 24 h post-thawing was significantly higher for cells frozen in SYF, CV, and Hypo media, but not for cells cryopreserved in PSC, CS10, CS5, or SCB media (Figure 3E). When the data are analyzed for cell recovery, defined as a percentage of viable cells to the initial number of frozen cells, there were no significance differences in cell recovery immediately upon thawing for any of the media (Figure 3F), but at 24 h post-thawing the DMSO-free media, CV and Hypo, had significantly lower percentages of live recovered cells than the best-performing medium, PSC (Figure 3G). While the performance of CV and Hypo were not significantly different from each other by this measure, Hypo was significantly poorer than all the DMSO-containing cryopreservation media, while the DMSO-free CV media was not statistically different from SCB and SYF media, which contain 10% DMSO (v/v) (Supplementary Table S2).

**FIGURE 4:**
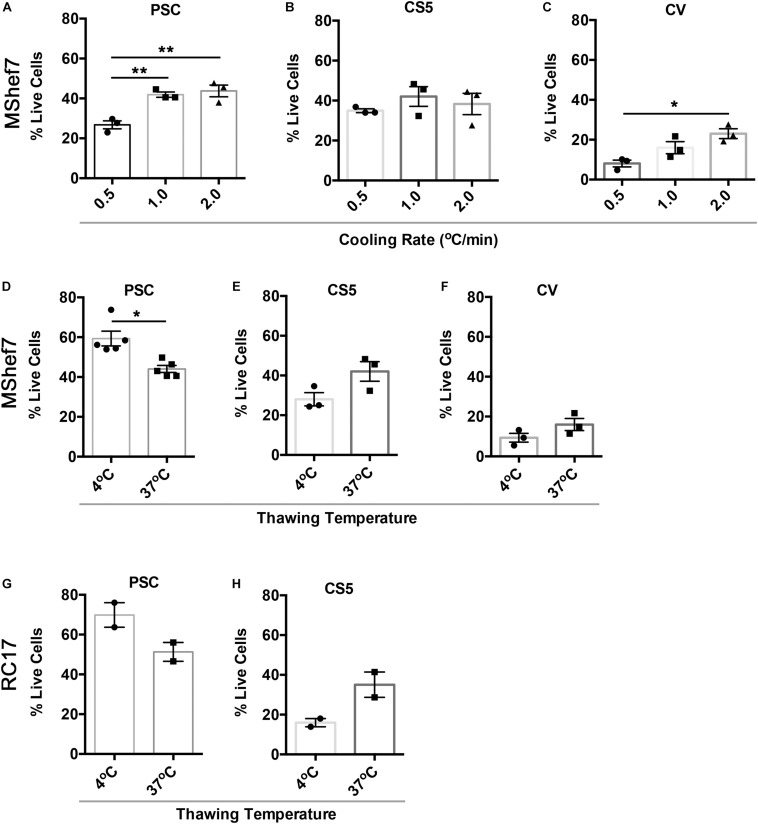
Cell recovery at different cooling rates and thawing conditions. Percentage of live attached cells recovered 24 h after thawing for cells cryopreserved at different cooling rates, 0.5, 1.0, and 2.0°C per minute, in PSC **(A)**, CS5 **(B)**, or CV **(C)** cryopreservation media in three independent experiments (*n* = 3). *p < 0.05, **p < 0.01, one-way ANOVA with Tukey’s multiple comparisons test. **(D–F)** Cell recovery at 24 h post-thawing for mDA cells frozen in PSC (*n* = 5), CS5 (*n* = 3), or CV (*n* = 3) cryopreservation media and thawed at 4 or 37°C in three to five independent experiments. *p < 0.05, unpaired *t*-test with Welch’s correction. **(G,H)** Cell recovery of RC17-derived mDA cells at different thaw rates (37 versus 4°C) in PSC and CS5 cryopreservation medium (*n* = 2 experimental replicates).

Since PSC was the best-performing cryopreservation medium in the head-to-head comparison (Figure 3D), we attempted to further improve cell recovery by investigating three different cooling rates and two thawing conditions. We also included CS5 and CV media, which contained 5% DMSO and 0% DMSO, respectively, since the presence of DMSO could affect downstream differentiation (Pal et al., 2012). For PSC media, 1 and 2°C/min were significantly better than 0.5°C/min at 24 h post-thawing, while there were no significant differences for cells frozen in CS5 medium (Figures 4A,B). For the DMSO-free CV medium, the fastest freezing rate of 2°C/min was significantly better than the slowest rate of 0.5°C/min (Figure 4C). Although it is common practice to thaw cells rapidly in a warm water-bath, we found that slow thawing in cool conditions (10 min at 4°C) was significantly better than fast thawing at 37°C for cells frozen in PSC media (Figure 4D). However, mDA neural progenitor cryopreserved in CS5 or CV media did not show significant differences between the slow and rapid thawing conditions (Figures 4E,F). mDA neural progenitor cells differentiated from another hESC line, RC17, also exhibited excellent survival 24 h post-thawing after cryopreservation in PSC medium (Figure 4G). In agreement with the MShef7 data, slow thawing at 4°C was equivalent, if not superior, to rapid thawing at 37°C for RC17-derived mDA neural progenitor cells frozen in PSC medium (Figure 4G). Cells frozen in the 5% DMSO medium, CS5, did not recover as well as cells cryopreserved in PSC medium; however, there was a trend toward better survival in the rapid 37°C thawing condition for mDA neural progenitor cells differentiated from both MShef7 and RC17 cell lines (Figures 4E,H).

**FIGURE 5:**
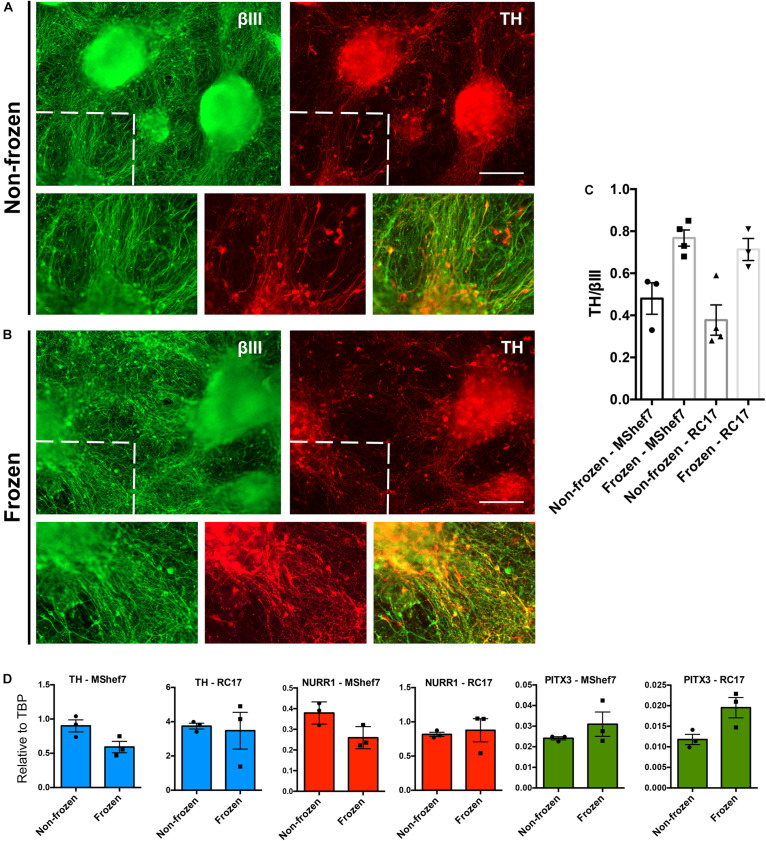
Marker analysis of continuously differentiated mDA neurons versus neurons that were cryopreserved at day 16 as mDA neural progenitor cells. Immunofluorescence staining for the pan-neuronal marker, βIII-tubulin (βIII), and dopaminergic marker, tyrosine hydroxylase (TH), at day 45 of differentiation of MShef7 hESCs without freezing **(A)** or with freezing **(B)** at day 16. Zoomed-in areas designated by dashed lines. Scale bars, 220 μm. **(C)** Image quantification of TH immunostaining as a fraction of βIII-tubulin immunostaining for non-frozen and frozen mDA neurons differentiated from MShef7 and RC17 hESC lines (*n* = 1 experimental replicate for each cell line, three to four images per condition). **(D)** RT-qPCR gene expression analysis of mDA markers *TH*, *NURR1*, and *PITX3* at day 42 of differentiation from MShef7 and RC17 hESC lines (*n* = 3 experimental replicates).

Next we directly compared the differentiation potential of MShef7 and RC17-derived non-frozen cells to mDA neural progenitor cells frozen at day 16 in the optimized condition: PSC cryopreservation medium cooled at 1°C/min and thawed slowly at 4°C. At 45 days of DA neuronal differentiation, there were no gross differences in the production of neurons for cells frozen at day 16 when compared to non-frozen cells in terms of morphology or expression of the pan-neuronal marker βIII-tubulin and the DA marker, tyrosine hydroxylase (TH) (Figures 5A,B). Quantification of TH immunostaining with respect to βIII-tubulin expression for both MShef7 and RC17 hESC-derived neurons at day 45 of differentiation did not reveal any significant differences between non-frozen cells and cells frozen at day 16 of the protocol (Figure 5C). Gene expression analysis of *TH*, and mDA transcription factor markers, *NURR1* and *PITX3*, also did not reveal any significant differences between frozen and non-frozen mDA neural progenitor cells differentiated from both MShef7 and RC17 hESC lines (Figure 5D).

## Discussion

Human pluripotent stem cells are unique because they can produce cell types of all three germ layers in culture, maintain high chromosomal stability, and are practically immortal (Thomson et al., 1998; Takahashi et al., 2007). These properties make hESCs and iPSCs ideal for modeling disease in a dish, as well as providing an unlimited source of cells for regenerative medicine applications (Cohen and Melton, 2011; Tabar and Studer, 2014). Numerous protocols exist to differentiate hESCs/iPSCs into a diverse range of cell types, including many ectodermal and neuronal subtypes (Tchieu et al., 2017). Recent advances in differentiation of ventral mDA neurons have provided a significant opportunity to model PD in a dish, and it has accelerated the route to cell replacement therapies for this condition (Barker et al., 2017). Although current differentiation protocols can produce high yields of mDA neurons, cell line-to-cell line differences continue to exist, as well as other sources of variation due in part to the complexity of the protocol (Nolbrant et al., 2017). It is well described that different hESC and iPSC lines have variable potency of directed differentiation into neurons and other cell types (Wu et al., 2007; Osafune et al., 2008; Hu et al., 2010). One solution to this is to produce a quality-controlled cryopreserved bank of mDA neural progenitor cells from which all experiments are initiated. This is a similar concept to the proposed CryoPause methodology where banks of hESCs or iPSCs are cryopreserved at one passage in a ready-to-differentiate format, without the need for expansion (Wong et al., 2017). Here we propose that mDA neural progenitor cells, frozen in a ready-to-use format, will significantly improve the reproducibility of disease modeling, and could facilitate efforts toward cell replacement therapy. A cryopreserved transplantation-ready mDA cell product would provide a critical opportunity for quality control measures, such as efficacy testing and safety assessments. The feasibility of a “poised” partially differentiated cell product was demonstrated when frozen iCell DopaNeurons were shown to be capable of rescuing the 6-OHDA lesion rat model of PD (Wakeman et al., 2017).

Since there is often significant cell loss associated with freezing and thawing of cells, we took a systematic approach to examine multiple aspects of the cryopreservation process in order to maximize the yield of viable mDA neural progenitor cells. To this end, we identified six commercially-available cryopreservation media to test against each other (Table 1), and investigated a clinical-grade 4°C hibernation medium, HypoThermosol^®^ (Hypo), which has been used to cryopreserve cells in the literature (Baust et al., 2000). We investigated different cooling rates with the VIA Freeze^TM^ Duo controlled-rate freezer, and compared rapid versus slow thawing of cells (Figure 2). We focused our cryopreservation experiments on day 16 of the mDA protocol, since this is the day that we lift the differentiating cells for re-plating, and this is the optimal stage of maturity for transplantation into the rat 6-OHDA lesion model of PD (Kirkeby et al., 2012). We first investigated the addition of a selective Rho-associated coiled-coil kinase (ROCK) inhibitor, Y27632, and the supplement Revitacell^TM^ that contains a ROCK inhibitor, to the recovery medium. We found that both agents significantly improved cell survival and cell recovery in the first 24 h after thawing (Figures 3A,B). These observations are in agreement with published work demonstrating that inhibition of ROCK in the first 12 h after passaging or thawing of cells prevents apoptosis in a number of cell types including hESCs and neurons (Watanabe et al., 2007; Lingor et al., 2008).

Surprisingly, there were no significant differences in cell viability immediately after thawing cells for any of the cryopreservation media, including the hibernation medium, Hypo (Figure 3C). Differences only emerged when cell viability and recovery were investigated at 24 h post-thawing (Figure 3D). At this time-point, Hypo was the worst-performing cryopreservation media, followed by SYF and CV, while the best-performing media, PSC, was not significantly different to freshly passaged cells (Figure 3D). The observations of cell viability at 0 and 24 h post-thawing are in agreement with data describing cryopreservation-induced delayed onset of cell death (Baust et al., 2000, 2001, 2009). Quantification of apoptosis in the first 48 h after thawing MDCK cells identified the highest rates of apoptosis to be between 12 and 24 h (Baust et al., 2001). This work and our observations strongly indicate that measuring cell viability and total cell recovery at 24 h post-thawing, not immediately upon thawing, is essential to evaluate the quality and efficiency of a cryopreservation process. Furthermore, an indirect, non-invasive measure of the efficiency of a cryopreservation protocol for adherent cells can be inferred from the number of floating cells at 24 h post-thawing (Figure 3E). This identified Hypo, CV, and SYF as significantly poorer than other cryopreservation media for mDA neural progenitor cells. Although DMSO-free CV medium was the worst-performing cryopreservation medium tested, it was consistently better than Hypo by all measures at 24 h post-thawing. DMSO is a highly effective cryopreservation agent, but other solutes, such as glycerol, can provide a measure of cryoprotection (Lovelock and Bishop, 1959).

Varying the cooling rate resulted in significant differences in 24 h post-thaw cell recovery for PSC and CV cryopreservation media, where faster cooling rates (1 and 2°C/min) were generally better than a slow cooling rate of 0.5°C/min (Figures 4A,C). Varying the cooling rate for cells frozen in CS5 media did not result in any significant differences in cell viability (Figure 4B). Slow cooling rates are known to avoid intracellular ice crystal formation in part by allowing the cells sufficient time to lose water (Mazur, 1963); however, excessively slow cooling may result in DMSO toxicity. Optimal cooling rates are dependent on cell type, but also cryoprotectant as we have seen here. Some cryoprotectants containing extracellular solutes will enhance cell dehydration, therefore allowing sufficient dehydration at more rapid cooling rates. Other solutes may be more toxic, and therefore cells will be more sensitive to slower cooling rates where cells remain in the cryoprotectant longer before reaching a cryopreserved state (Fuller, 2004).

We next investigated rapid versus slow thawing conditions. Cryopreserved cells are typically thawed quickly in a 37°C water-bath, and slow thawing is usually considered undesirable, which is supported by limited experimental data from the 1970s (Harris and Griffiths, 1977; Akhtar et al., 1979). However, warming rates for cryopreserved mammalian somatic cells were recently re-visited, and no impact of thawing conditions could be identified for cells cooled slowly at a rate of 1°C/min (Baboo et al., 2019). Rapid thawing was only beneficial for cells that were frozen quickly at 10°C/min or faster. Such rapid rates of cooling result in only partial ice crystal formation, and rapid thawing could rescue some damage due to re-crystallization on warming. If cells are cryopreserved slowly in optimal conditions, then the warming rate had little impact on viability as ice crystals fully develop during the cooling process (Baboo et al., 2019). In agreement with these data, we did not observe a significant improvement in cell viability or total cell recovery for cells thawing quickly in a 37°C water-bath compared to cells thawed slowly in air at 4°C. In fact, slow thawing at 4°C for MShef7-derived mDA neural progenitor cells was significantly better than rapid thawing (Figure 4D). An additional reason why slow thawing might be beneficial could be the non-uniform nature of thawing frozen liquids, which could be exaggerated during rapid thawing. Ice does not melt uniformly at the microscale, and it can melt in some places, and re-freeze in other places, leading to pockets of ice re-crystallization that can damage cells. Slow thawing could reduce these micro-fluctuations. Recent studies with HepG2 cells suggested slow thawing up to −10°C, and then rapid thawing from −10 to 4°C is beneficial due to the reduction of such fluctuations (Kilbride et al., 2017). Finally, rapid thawing may not be beneficial due to the potential for increased toxicity of DMSO, especially if temperatures approach 37°C before wash-out (Morris et al., 2016). Controlled-rate thawing to 4°C, followed by immediate dilution of cells in cold medium prior to centrifugation, could minimize potential toxic effects of DMSO.

We next directly compared the neuronal differentiation potential of MShef7 and RC17-derived cryopreserved mDA neural progenitor cells to non-frozen cells up to 45 days of differentiation. There were no gross morphological differences nor quantitative differences in TH immunostaining between the mDA neurons produced from frozen versus non-frozen cells (Figures 5A–C). Furthermore, we did not observe any significant differences in gene expression of DA markers, *TH*, *NURR1*, and *PITX3* for frozen versus non-frozen DA neurons differentiated from RC17 and MShef7 hESCs (Figure 5D).

This study provides the first systematic investigation of cryopreservation conditions for human mDA neural progenitor cells. We found that assessment of cell recovery at 24 h post-thawing, and not immediately upon thawing, was essential to distinguish between good and poor cryopreservation conditions. Furthermore, we challenge the notion that rapid thawing of cells is necessarily better than slow thawing conditions. Indeed, non-linear cooling and warming conditions could further improve the cell viability and total cell yield of human mDA neural progenitor cells, and this warrants further investigation. Other parameters that can be investigated include cell density, total freezing volume, and cryovial type (material, wall thickness), which were all kept constant in this study. High-quality cryopreserved mDA neural progenitor cells with high post-thaw viability will be a valuable resource for the neuroscience research community, and could facilitate the efforts toward cell replacement therapy for Parkinson’s by providing a time window for safety testing and assessment of efficacy.

## Data Availability Statement

The datasets presented in this study can be found in online repositories. The name of the repository and digital object identifier can be found below: LabArchives https://doi.org/10.25833/8a4w-4y22.

## Author Contributions

TK designed the study and wrote the manuscript. ND performed the experiments and data analysis. KSD and MC provided reagents and optimized protocols. PK and GJM contributed to study design, data analysis, and loan of VIA Freeze^TM^ Dou controlled-rate freezer. All authors edited and approved the manuscript.

## Conflict of Interest

PK and GJM are employed by Cytiva, United Kingdom. The remaining authors declare that the research was conducted in the absence of any commercial or financial relationships that could be construed as a potential conflict of interest.

## Supplementary Material

Supplementary Table 1 Catalog number for all reagents.

Supplementary Table 2 *p*-Values and significance calls for all statistical tests performed.
